# HIF2α‐induced upregulation of *RNASET2* promotes triglyceride synthesis and enhances cell migration in clear cell renal cell carcinoma

**DOI:** 10.1002/2211-5463.13570

**Published:** 2023-02-12

**Authors:** Yanmei Quan, Jun Dai, Sian Zhou, Lingyi Zhao, Lixing Jin, Yijing Long, Siwei Liu, Yanqin Hu, Yue Liu, Juping Zhao, Zhide Ding

**Affiliations:** ^1^ Department of Histology, Embryology, Genetics and Developmental Biology, Shanghai Key Laboratory for Reproductive Medicine Shanghai Jiao Tong University School of Medicine China; ^2^ Department of Urology, Ruijin Hospital Shanghai Jiao Tong University School of Medicine China; ^3^ Department of Clinical Medicine Shanghai Jiao Tong University School of Medicine China

**Keywords:** cancer stages, clear cell renal cell carcinoma (ccRCC), HIF2α, RNASET2, triglycerides synthesis

## Abstract

Clear cell renal cell carcinoma (ccRCC), the most common malignant subtype of renal cell carcinoma, is characterized by the accumulation of lipid droplets in the cytoplasm. *RNASET2* is a protein coding gene with a low expression level in ovarian cancers, but it is overexpressed in poorly differentiated neuroendocrine carcinomas. There is a correlation between *RNASET2* upregulation and triglyceride expression levels in human serum but is unknown whether such an association is a factor contributing to lipid accumulation in ccRCC. Herein, we show that *RNASET2* expression levels in ccRCC tissues and cell lines are significantly higher than those in both normal adjacent tissues and renal tubular epithelial cells. Furthermore, its upregulation is associated with increases in ccRCC malignancy and declines in patient survival. We also show that an association exists between increases in both cytoplasmic lipid accumulation and HIF‐2α transcription factor upregulation, and increases in both *RNASET2* and triglyceride expression levels in ccRCC tissues. In addition, DGAT1 and DGAT2, two key enzymes involved in triglyceride synthesis, are highly expressed in ccRCC tissues. By contrast, *RNASET2* knockdown inhibited their expression levels and lowered lipid droplet accumulation, as well as suppressing *in vitro* cell proliferation, cell invasion, and migration. In conclusion, our data suggest HIF2α upregulates *RNASET2* transcription in ccRCC cells, which promotes both the synthesis of triglycerides and ccRCC migration. As such, *RNASET2* may have the potential as a biomarker or target for the diagnosis and treatment of ccRCC.

## Graphical abstract

In normal kidney, HIF2α exists at low levels in the cytoplasm under normal oxygen conditions. However, in *VHL*‐deficient clear cell renal cell carcinoma (ccRCC), HIF2α accumulates in the cytoplasm under hypoxia and is then transported into the nucleus. HIF2α‐induced *RNASET2* upregulation increases *DGAT* expression, thereby promoting triglyceride synthesis and cell migration in ccRCC.

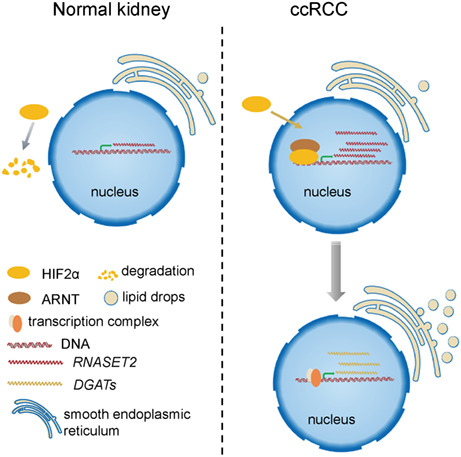

**Abbreviations**
ANT: adjacent normal tissue
CCLE: Cancer Cell Line Encyclopedia
ccRCC: clear cell renal cell carcinoma
chRCC: chromophobe cell renal carcinoma
ChIP: chromatin immunoprecipitation
DFS: disease‐free survival
DGAT: diacylglycerol O‐acyltransferase
GEPIA: gene expression profiling interactive analysis
H&E staining: hematoxylin–eosin
IHC: staining and immunohistochemistry
KEGG: Kyoto Encyclopedia of Genes and Genomes
pRCC: papillary renal cell carcinoma
RNASET2: RNase T2
RTCA: real‐time cell analysis
TCGA: The Cancer Genome Atlas
TEM: transmission electron microscopy
TG: triglyceride

According to the Global Cancer Observatory in 2020, the incidence of kidney cancer in males ranks tenth among all cancers in the world. Renal cell carcinoma (RCC) is the most malignant type of kidney cancer and its incidence is recently increasing worldwide [1]. Four main subtypes of RCC are identifiable based on their histopathological classification. They include clear cell renal carcinoma (ccRCC), chromophobe cell renal carcinoma (chRCC), and papillary renal cell carcinoma (pRCC), and collect duct carcinoma. ccRCC is the most common subtype of RCC, which accounts for more than 70% of the total cases, usually accompanying a high rate of metastasis and recurrence [1, 2]. Pathological analyses show that ccRCC arises from the epithelial cells lining the lumen of the proximal convoluted tubules in the kidney. Increases in the hematoxylin–eosin (H&E) staining are attributable to rises in cytoplasmic lipid and glycogen content, which caused the cytoplasm to become transparent [3]. Notably, although ccRCC presents a variety of symptoms in its early stages, it often has an asymptomatic clinical manifestation [3, 4]. Its clinical diagnosis is hindered by the lack of specific biomarkers, which accounts for its poor prognosis [5]. Therefore, there is an urgent need to identify novel‐targeted biomarkers that will enable earlier diagnosis and treatment of ccRCC.

*RNASET2* is a single‐copy gene located at chromosome 6q27, a region related to cell malignancy and chromosomal rearrangement [6]. It is a secreted protein that is the only member of the Rh/T2/S glycoprotein family expressed in humans. It is present in the cytoplasm outside the perinuclear region, where it colocalizes with endoplasmic reticulum markers and cis/trans‐Golgi [7, 8]. RNASET2 has diverse functions, including scavenging ribonucleic acid, degradation of self‐RNA, modulating host immune responses, etc. [7, 8, 9, 10]. The adipose coexpression networks reveal a correlation between the expression of *RNASET2* in adipocytes and the content of triglycerides in humans [11]. Remarkably, RNASET2 exerts its ‘antitumor function’ independently of its ribonuclease activity [12]. Several studies documented that *RNASET2* plays a vital role as a tumor suppressor gene in the progression of ovarian tumors and melanomas. However, a recent study showed that *RNASET2* was downregulated in the early stage of gastric adenocarcinoma compared with adjacent noncancerous or normal gastric mucosa tissues, but the knockdown or knockout of *RNASET2* did not significantly promote gastric adenocarcinoma cell growth [13]. Meanwhile, Uccella *et al*. [14] reported that the RNASET2 protein was significantly highly expressed in poorly differentiated neuroendocrine neoplasms of the lung compared with that in well‐differentiated tissues. It suggested that *RNASET2* may instead have different biological effects that are either tumor type‐dependent or related to their stage of progression. Besides, the role of RNASET2 in disrupting lipid metabolism warranted clarification in ccRCC.

In the current study, we show that there are stage‐dependent variations in the *RNASET2* expression level relative to their invariance in normal kidney tissue. This difference prompted us to clarify if: (a) an association exists among *RNASET2* expression levels and ccRCC oncogenesis; (b) dysfunctional lipid metabolism is an underlying mechanism of this pathological condition.

## Results

### Selective *RNASET2* upregulation in clear cell renal cell carcinoma

**Fig. 1:**
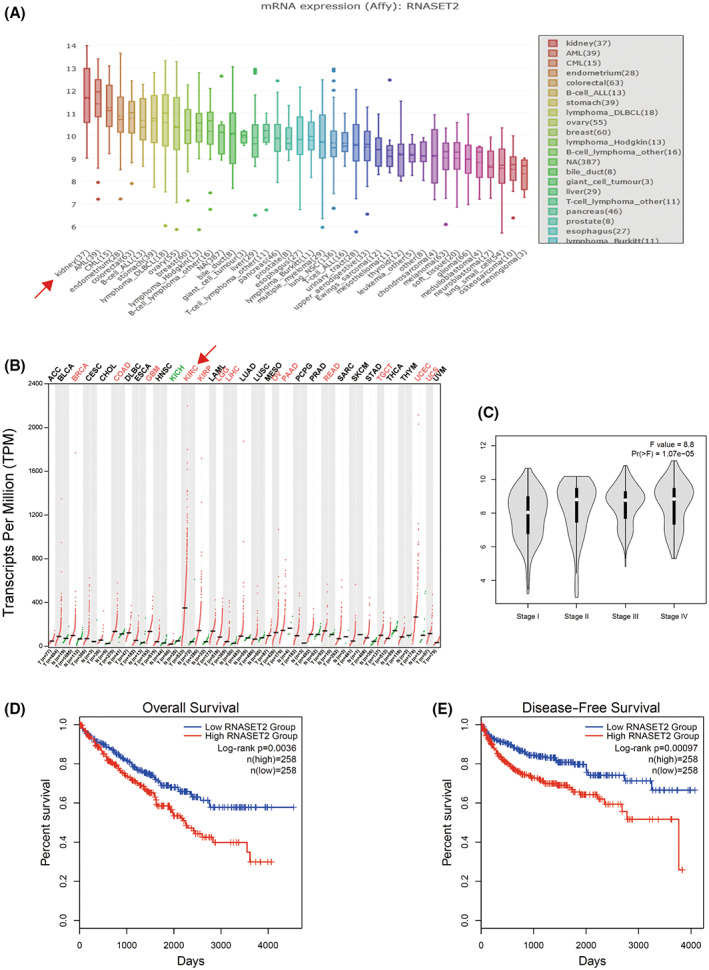
*RNASET2* expression is higher in clear cell renal cell carcinoma. (A) CCLE database shows that *RNASET2* mRNA is overexpressed in kidney cancer cell lines; (B) *RNASET2* expression is significantly higher in ccRCC tissues compared with that in normal kidney tissues; (C) *RNASET2* expression in different stages of ccRCC; (D) Overall survival of ccRCC patients with different expression levels of *RNASET2* (normalized by *ACTB*), red line: high expression, blue line: low expression; (E) disease‐free survival (DFS) of *RNASET2* expression in patients using TCGA datasets (normalized by *ACTB*).

Although several studies reported that *RNASET2* is a tumor suppressor gene in ovarian cancer and melanoma [12, 15, 16, 17], RNASET2 is overexpressed in poorly differentiated rather than in well‐differentiated neuroendocrine neoplasms of the lung [14]. This disconnect prompted us to speculate that *RNASET2* may have diverse expression levels and functions in different tumors. The Broad Institute controls a cancer research project entitled the Cancer Cell Line Encyclopedia (CCLE), which originally focused on characterizing DNA copy numbers, DNA mutations, gene expression, and microRNA profiling, as well as assessing DNA methylation status, across nearly 1000 cell lines from over 30 kinds of cancerous tissues. These studies were appended with metabolite profiling in 2019 [18]. CCLE reveals that the expression of *RNASET2* is significantly higher in kidney cancer than that in other cancers (Fig. 1A). GEPIA2 (Gene Expression Profiling Interactive Analysis 2) developed from GEPIA has been a valuable resource for gene expression analysis based on tumor and normal samples from the TCGA (The Cancer Genome Atlas) and GTEx (Genotype‐Tissue Expression) databases [19]. Since ccRCC is the main type of tumor in kidney malignancy, we analyzed using TCGA datasets at GEPIA2 and found that *RNASET2* gene expression was significantly higher in ccRCC tissues compared with that in other cancers (Fig. 1B). Moreover, the expression of *RNASET2* mRNA increased significantly in the advanced stages of ccRCC (Fig. 1C).

We then assessed the survival duration of patients with different *RNASET2* expression levels using TCGA datasets at GEPIA2. Overall survival analysis showed that ccRCC patients with high *RNASET2* expression levels had survival times that were significantly shorter than those ccRCC patients with lower *RNASET2* expression levels (Fig. 1D). Disease‐free survival (DFS) analyses revealed that the *RNASET2* expression level was correlated with DFS of ccRCC patients (Fig. 1E).

**Fig. 2:**
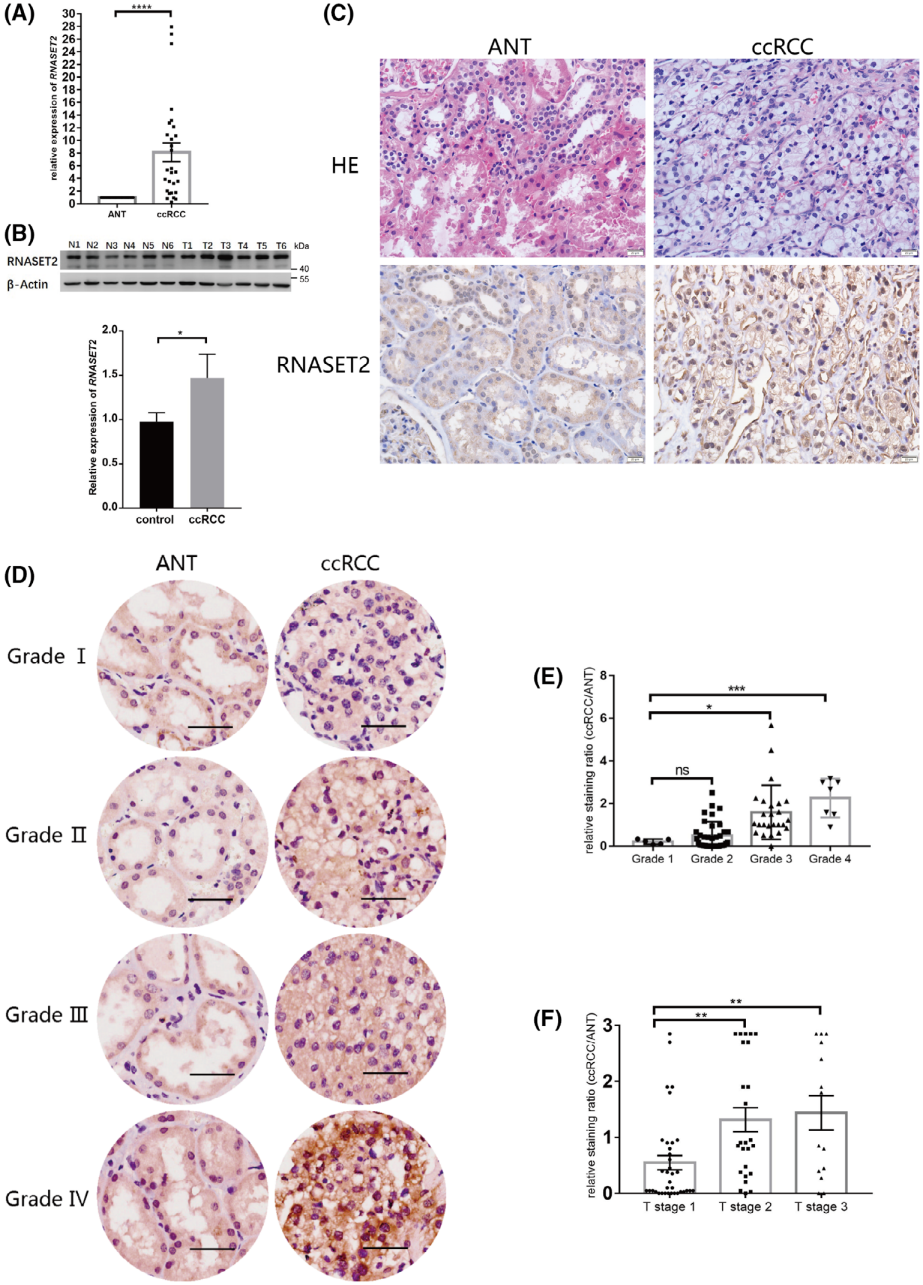
*RNASET2* expression in ccRCC exceeds that in ANT. (A) The relative mRNA expression level of *RNASET2*, *n* = 28; (B) The relative protein expression level of RNASET2 (upper, the results of western blot, N1 ~ N6: adjacent normal tissues, T1 ~ T6: ccRCC tissues; lower, analyses of western blot by gray value), *n* = 28; (C) Hematoxylin–eosin (H&E) staining and immunohistochemistry (IHC) of RNASET2 in ccRCC and ANT, scale bars: 50 μm; (D) IHC chip of RNASET2 in ccRCC and ANT, scale bars: 50 μm; (E) The relative intensity in the IHC chip of RNASET2 expression level in different ccRCC grades, Number of different grades of cases: *n*_Grade 1_ = 5, *n*_Grade 2_ = 37, *n*_Grade 3_ = 25, *n*_Grade 4_ = 7; (F) The relative intensity in the IHC chip of RNASET2 expression level in different ccRCC T stages, Number of different stages of cases: *n*_stage 1_ = 35, *n*_stage 2_ = 25, *n*_stage 3_ = 14. Values in bar graphs are the mean with SD. Statistical analysis was performed using the Student's *t*‐test (A and B) and one‐way ANOVA (E and F). *P < 0.05, **P < 0.01, ***P < 0.001, ****P < 0.0001; ns, not significant.

To confirm the actual expression level of *RNASET2* in cancer tissues obtained from the ccRCC patients in our affiliated hospital, real‐time quantitative RT‐PCR and western blot analyses were applied, respectively, to detect both the *RNASET2* mRNA expression levels and their corresponding protein contents in ccRCC tissues. In 28 ccRCC cases, their expression levels were compared with the corresponding adjacent normal tissues (ANT). The results showed that the *RNASET2* expression levels were significantly higher in ccRCC tissues both at both the mRNA and protein expression levels (Fig. 2A,B). IHC analysis using an RNASET2 antibody confirmed higher RNASET2 protein expression levels than those in the ANT tissues (Fig. 2C). These results agreed with those obtained from the analyses of the TCGA datasets (Fig. 1B). On the contrary, an analysis was performed of an immunohistochemical chip (IHC chip) including 74 pairs of ccRCC tissues and their corresponding ANT. The results showed that the levels of RNASET2 protein were much higher in the poorly differentiated (high‐grade) ccRCC (Fig. 2D,E). Then, we determined whether there is a correlation between the RNASET2 protein expression level and several key factors relevant to the patient's health status, including age, gender, tumor size, tumor grades, and clinical tumor node metastasis (cTNM) stages. The results revealed that the RNASET2 protein expression level significantly correlated with the ccRCC grades and Tumor stages (T stages) of ccRCC patients (Table 1, Fig. 2E,F). In other words, increases in RNASET2 protein expression levels correlated with both rises in ccRCC malignancy and *RNASET2* mRNA upregulation. Thus, these agreements strongly suggest that RNASET2 may be a relevant molecular marker for both diagnosis and improved therapeutic management of ccRCC disease.

**Table 1:** RNASET2 expression in 74 pairs of clear cell renal cell carcinoma compared with adjacent normal tissues.

	RT2 expression in tumor		*N*	*P*‐value
	Low	High		
Age (year)				
≤ 65	35	15	50	0.599
> 65	13	10	23	
Gender				
Male	34	15	49	0.794
Female	15	10	25	
Tumor size				
≤ 5 cm	18	7	25	0.134
> 5 cm	31	18	49	
Grade				
I	3	0	3	< 0.001 (***)
II	31	4	35	
III	11	17	28	
IV	4	4	8	
T stage				
1	29	8	37	0.004 (**)
2	14	10	24	
3	6	7	13	
N stage				
0	46	22	68	0.959
1	1	1	2	
2	2	1	3	
M stage				
0	49	24	73	0.059
1	0	1	1	
cTNM stage				
1	27	6	33	0.008 (**)
2	14	9	23	
3	6	8	14	
4	2	2	4	

** *P* < 0.01

*** *P* < 0.001.

### Upregulation of DGAT1 and DGAT2 expression levels in ccRCC tissues

**Fig. 3:**
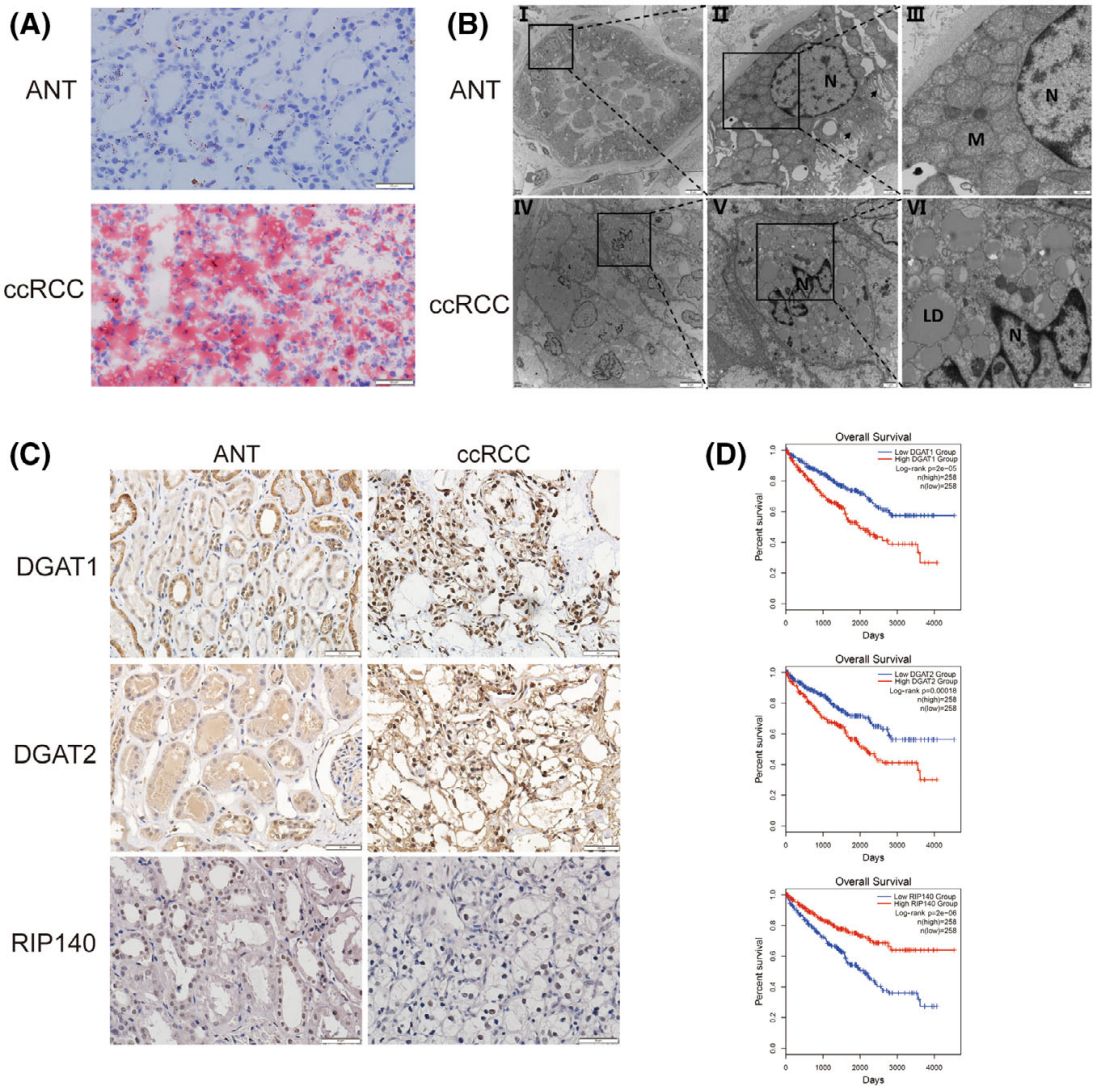
High expressions of DGAT1 and DGAT2 and low expression of RIP140 in ccRCC accompanied lipid droplet accumulation. (A) Oil Red O staining monitored cytoplasmic lipid droplet accumulation in the ccRCC, scale bars, 50 μm; (B) TEM evaluation of cytoplasmic lipid droplets that accumulated in the ccRCC, M: mitochondria; N: nucleus; LD: lipid droplet; scale bars (from left to right): 5 μm, 1 μm, and 500 nm; (C) IHC analysis compared DGAT1, DGAT2, and RIP140 expression levels in ccRCC with their corresponding values in ANT, scale bars: 50 μm; (D) Analysis of the overall survival curves of *DGAT1*, *DGAT2*, and *RIP140* expressions base on the TCGA database (normalized by endoplasmic reticulum marker *CANX* for both *DGAT1* and *DGAT2*, *ACTB* for *RIP140*), red line: high expression; blue line: low expression.

Abnormal lipid accumulation is the most significant change in a series of different ccRCC phenotypes. On the contrary, the *RNASET2* expression levels correlated significantly with the content of triglycerides in humans [11]. We next determined whether the triglyceride synthesis was promoted in ccRCC. Differences in Oil Red O staining intensity showed that the lipid content was much higher in ccRCC tissues than that in its corresponding ANT control (Fig. 3A). Furthermore, lipid droplets completely filled the cytoplasmic area in ccRCC tissue, whereas TEM analysis showed that the mitochondrial density content waned (Fig. 3B). In general, lipid accumulation in the cells is a pathophysiological process that reflects an imbalance between lipid synthesis and lipolysis. Diacylglycerol acyltransferases, DGAT1 and DGAT2, are key enzymes mediating triglyceride (TG) synthesis. They catalyze the esterification of fatty acid‐CoA (FA‐CoA) and promote TG synthesis from diacylglycerol (DAG). In this study, both DGAT1 and DGAT2 were highly expressed in ccRCC tissue compared with those in the corresponding ANT (Fig. 3C). Notably, according to the analysis of the GEPIA2 database, ccRCC patients with high *DGAT1* or *DGAT2* expression (normalized by endoplasmic reticulum marker *CANX*) usually had a shorter survival time (Fig. 3D). On the contrary, cytoplasmic RIP140 protein is one of the critical factors relevant to triglyceride metabolism and it can promote the lipolysis process [20]. Then, RIP140 protein was detected using IHC in ccRCC and ANT tissues. The results showed that RIP140 protein decreased significantly in ccRCC especially in the cytoplasm (Fig. 3C), indicating that the lipolysis process in ccRCC might be inhibited. Moreover, according to the analysis of the TCGA database, a diminished expression of RIP140 content was also correlated with shorter survival of ccRCC patients (Fig. 3D). Therefore, it is presumed that tumor cells extended their survival in advanced stages by enhancing TGs synthesis and inhibiting lipolysis through suppressing fatty acid β‐oxidation.

### Silence of *RNASET2* downregulates TG synthesis, cell viability, cell invasion, and cell migration of *VHL*‐Deficient ccRCC cells

**Fig. 4:**
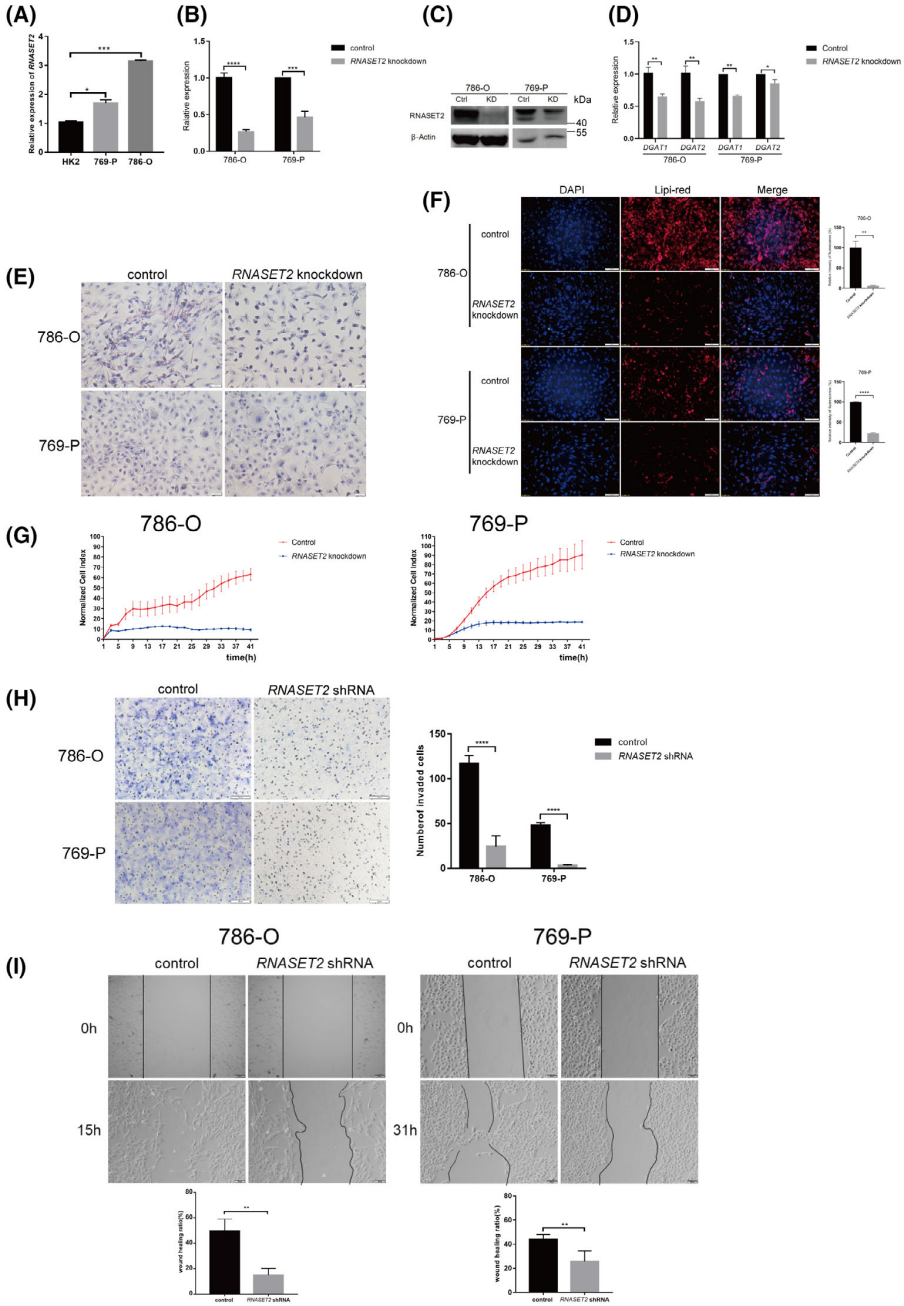
*RNASET2* expression influenced the expressions of lipids‐associated genes, cell proliferation, and cell migration. (A) mRNA expression level of *RNASET2* in HK‐2, 769‐P, and 786‐O cells, respectively, *n* = 3; (B, C) *RNASET2* was knocked down in ccRCC cells by *RNASET2* shRNA, *n* = 3; (D) *RNASET2* knockdown in ccRCC cells decreased both *DGAT1* and *DGAT2* expressions, *n* = 6; (E) Oil Red O staining, scale bars: 50 μm; (F) Assessment of lipi‐red staining, scale bars: 100 μm; (G) Real‐time cell analysis (RTCA) of cell proliferation showed that it was inhibited after *RNASET2* knockdown in ccRCC cells; (H) Transwell assay to evaluate cell invasion after *RNASET2* knockdown in ccRCC cells, scale bars: 100 μm, *n* = 6; (I) The scratch wound healing test showed that *RNASET2* deficiency inhibited the migration of ccRCC cells, scale bars: 100 μm, *n* = 6. Values in bar graphs are the mean with SD. Statistical analysis was performed using the Student's *t*‐test. *P < 0.05, **P < 0.01, ***P < 0.001, ****P < 0.0001.

Both high expression levels of *RNASET2* and TG synthesis‐related genes (*DGAT1* and *DGAT2*) in ccRCC prompted us to explore the relationship between them. Real‐time quantitative RT‐PCR assessed the mRNA expression levels of *RNASET2* in *VHL*‐deficient 786‐O and 769‐P ccRCC cells and human proximal tubular epithelial cells HK‐2 cells. *RNASET2* expression levels ranged from high to low levels in the following cells: 786‐O cells > 769‐P cells > HK‐2 cells (Fig. 4A). *RNASET2* shRNA plasmids were transfected into 786‐O or 769‐P cells followed by screening with 200 μm G418 (Fig. 4B,C). Both *DGAT1* and *DGAT2* expression levels were significantly reduced after *RNASET2* knockdown (Fig. 4D), whereas the expression levels were invariant of the lipolysis‐associated *RIP140*, *ABHD5*, and *ATGL* genes (Fig. S1A). The results of Oil Red O staining showed that the intracellular lipid droplets decreased after *RNASET2* knockdown in 786‐O and 769‐P cells (Fig. 4E), and the lipi‐red fluorescence staining was similar (Fig. 4F). To confirm the effect of *RNASET2* expression on cell proliferation, real‐time cellular analysis (RTCA) using xCELLigence® Real‐Time Cell Analyzer was applied. The results showed that cell proliferation was inhibited after *RNASET2* knockdown in ccRCC cells (Fig. 4G). The traditional CCK‐8 assay also showed that cell proliferation was significantly inhibited in 786‐O cells after *RNASET2* knockdown (Fig. S1B). Therefore, these results directly indicated that *RNASET2* deficiency inhibited ccRCC cell growth *in vitro*.

In addition, cell invasion and migration are two important indicators for evaluating tumor progression. Both the transwell assay and the scratch wound healing assay showed that the *RNASET2* knockdown in ccRCC cells inhibited cell invasion and migration (Fig. 4H,I). Furthermore, the expression was detected of the metastasis‐associated gene (*MTA2*), which is a reflection of cell migratory activity [21]. It was evaluated with the scratch wound healing assay in 786‐O cells. The result showed that *MTA2* expression level was much lower in *RNASET2* knockdown 786‐O cells than that in control cells suggesting that *RNASET2* knockdown impaired cell migration (Fig. S1C). Taken together, these results suggest that *RNASET2* expression is essential for TG synthesis, and the maintenance of cell proliferation, cell invasion, and migration in *VHL*‐deficient ccRCC Cells.

To further confirm the roles of *RNASET2* expression in mediating control of the expression levels of the lipids‐associated genes, *RNASET2* coding sequences were transfected into 769‐P ccRCC cells. The results showed that *RNASET2* overexpression significantly upregulated the key enzymes mediating triglyceride synthesis: *DGAT1* and *DGAT2* but not the lipolysis‐associated gene *RIP140* (Fig. S1D). Meanwhile, RTCA measurements showed that cell proliferation was not influenced (Fig. S1E). These results strongly suggest that *RNASET2* mainly influences triglyceride synthesis rather than lipolysis in ccRCC.

### HIF2α directly regulates *RNASET2* transcription in ccRCC

**Fig. 5:**
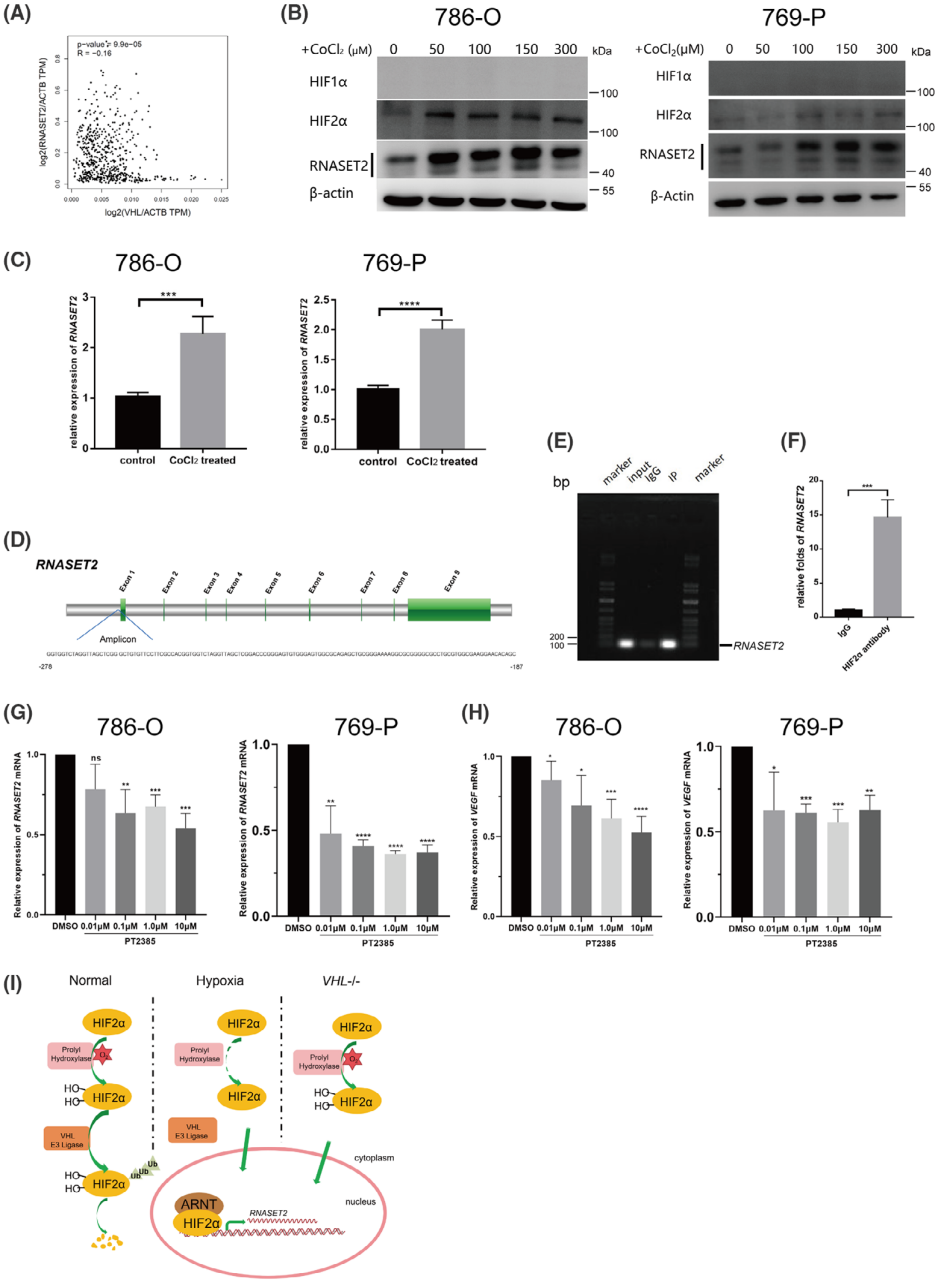
HIF2α directly interacted with *RNASET2* in ccRCC cells. (A) TCGA database analysis shows that there is a negative correlation between *RNASET2* and *VHL* expression levels, *P* = 9.9e‐05, *R* = −0.16; (B) Following exposure to CoCl_2_ for 24 h, both HIF2α and RNASET2 proteins were highly upregulated in 786‐O and 769‐P cells with HIF1α deficiency; (C) The *RNASET2* mRNA expression level increased significantly in 786‐O and 769‐P cells after exposure to 150 μm CoCl_2_ for 24 h (reference gene: *ACTB*), *n* = 6; (D) Sequence of ChIP‐PCR amplicon corresponding to *RNASET2* gene structure (green fragments: exons); (E, F) Fragments pulldown with HIF2α antibody by chromatin immunoprecipitation (ChIP) was analyzed by regular PCR and quantitative real‐time PCR, *n* = 3; (G) *RNASET2* transcription in 786‐O and 769‐P cells after exposure to different doses of PT2385 for 24 h (control: DMSO, reference gene: *ACTB*), *n* = 6; (H) *VEGF* transcription in 786‐O and 769‐P cells after exposure to different doses of PT2385 for 24 h (control: DMSO, reference gene: *ACTB*), *n* = 6; (I) A schematic representation showing the regulatory mechanism of *RNASET2* transcription in ccRCC. Values in bar graphs are the mean with SD. Statistical analysis was performed using the Student's *t*‐test (C and F) and one‐way ANOVA (G and H). *P < 0.05, **P < 0.01, ***P < 0.001, ****P < 0.0001; ns, not significant.

VHL/HIFα pathway is one of the most important regulatory pathways in ccRCC. The tumor suppressor VHL targets HIF1α and HIF2α earmarked for proteasomal degradation [22]. Both HIF1α and HIF2α are hypoxia‐induced transcription factors (HIF) in human cells. We evaluated whether HIF1α regulates RNASET2 transcription in ccRCC. Using the TCGA datasets, *RNASET2* mRNA expression was negatively correlated with *VHL* mRNA expression in ccRCC (Fig. 5A). The two main HIFα isoforms, HIF1α and HIF2α, have opposing effects on renal cell carcinoma biology, with HIF1α acting as a tumor suppressor, whereas HIF2α is an oncogene [23]. ccRCC tumors that only express HIF2α have higher proliferation rates than those expressing both HIF1α and HIF2α [24]. HIF2α is a necessary key factor for the formation of ccRCC xenografts, whereas the knockdown of HIF1α enhances xenograft formation in cell lines expressing both HIF1α and HIF2α [25]. In the ccRCC 786‐O and 769‐P cell lines, which are both *VHL*‐deficient, they display intragenic deletions of HIF1α but express wild‐type HIF2α [25]. We found that the HIF2α protein accumulated in the ccRCC cases based on the results of western blot analysis while the HIF1α protein was deficient (Fig. S2). CoCl_2_ exposure is a generally accepted experimental model that simulates hypoxia and inhibition of HIFα hydroxylation, which results in its accumulation in the cytoplasm. The exposure of ccRCC cells to CoCl_2_, resulted in rises in the accumulation of both the HIF2α and the RNASET2 protein in ccRCC cells (Fig. 5B). Meanwhile, *RNASET2* mRNA expression also was significantly induced in ccRCC cells after exposure to 150 μm CoCl_2_ for 24 h (Fig. 5C). Thus, we speculated that *RNASET2* is the target gene of HIF2α. Then, chromatin immunoprecipitation‐PCR (ChIP‐PCR) was performed to determine whether HIF2α interacted with the promoter of *RNASET2*. We designed the specific PCR primers to amplify a span from −278 to −187 upstream of the transcription start site (TSS) of the *RNASET2* gene (Fig. 5D), which is generally considered the promoter region. ChIP‐PCR results showed that HIF2α combined directly with the *RNASET2* promoter (Fig. 5E,F). To functionally confirm the relationship between HIF2α and the *RNASET2* gene, we treated ccRCC cells with a HIF2α inhibitor PT2385, which inhibits the interaction between HIF2α and ARNT (also known as HIFβ) thus blocking the activity of HIF2α. After exposure to different concentrations of PT2385 for 24 h, *RNASET2* transcription was detected and the results showed that treatment with 0.1 μm PT2385 significantly decreased its expression in ccRCC cells (Fig. 5G). Accordingly, *VEGF*, the proven target of HIF2α, was also declined in parallel as expected (Fig. 5H). Therefore, these results indicated that the accumulated HIF2α induced *RNASET2* expression in ccRCC (Fig. 5I).

## Discussion

ccRCC, originating from the renal epithelial cells in the kidney cortex, does not only constitute the most frequent type of renal cancer, but it is also the second most prevalent tumor type listed in the urinary system. Its pathological identification includes that its cytoplasm is full of TGs and glycogens. With H&E staining, lipid droplets caused the cells to appear transparent [1], which was confirmed based on our results with Oil Red O staining. Moreover, our TEM analysis also revealed a high density of lipid droplets and few organelles in the cancerous cytoplasm. Due to the unique characteristics of cancerous tissue, until now there are no effective therapeutic procedures except surgery to delay its progression. One of the possible reasons for the inability to arrest its spread may stem from the huge lipid deposits in the cancerous cytoplasm [26, 27]. Despite this insight, there are no novel targets that can reverse ccRCC oncogenesis. If this void can be overcome, it will undoubtedly help in diagnosing and improving the therapeutic management of ccRCC and thereby prolong survival in the early stage of its development.

For tumor biology, RNASET2 is a multifunctional protein, which has dual‐directional roles in tumor development. Some studies showed that *RNASET2* plays a role as an antitumor gene in ovarian cancer and melanoma [12, 17, 28]. For example, *RNASET2* expression decreased both in ovarian tumors and melanoma and it was negatively correlated with the malignancy of these tumors [12, 29]. Moreover, the downregulated expression of *RNASET2* is generally associated with drug resistance in the treatment of ovarian cancer [30]. On the contrary, RNASET2 expression is significantly higher in poorly differentiated cancer cells than that in the well‐differentiated cancer cells in neuroendocrine neoplasms of the lung [14].

The current study provides suggestive evidence that *RNASET2* has the potential to become a new target for ccRCC diagnosis and therapy. In our study, the expression of *RNASET2* is significantly higher in ccRCC compared with that in the normal kidney based on TCGA dataset analyses, especially in the advanced stages of ccRCC, which was confirmed in ccRCC and ANT samples collected at our affiliated hospital. It should be emphasized that this expression pattern is somewhat different from prior reports on ovarian cancer research [12]. In addition, patients with high *RNASET2* expression in the ccRCC tissues usually had a poor prognosis compared with those with low *RNASET2* expression. Therefore, these indicate that *RNASET2* high expression in ccRCC tissues may be associated with shorter survival times in ccRCC patients. This inverse relationship is one piece of evidence supporting the notion that monitoring *RNASET2* expression levels may ultimately serve as a novel diagnostic and therapeutic target in a clinical setting.

There is other evidence suggesting the feasibility of RNASET2 serving as a target to improve the detection and therapeutic management of ccRCC. Haas *et al*. [11] reported that the high *RNASET2* expression in adipocytes was associated with the high content of TG in human serum. Such high levels also underlie the H&E‐stained pathological transparency of the ccRCC that results from their high TG content [1]. To clarify the influences of *RNASET2* on cell lipid metabolism, we detected the expressions of key genes that are relevant to controlling the TG synthesis and lipolysis pathway. DGAT1 and DGAT2 are two diacylglycerol acyltransferases, which catalyze the terminal and are only the committed step in TG synthesis using diacylglycerol and fatty acyl‐CoA. Moreover, DGAT1 is highly expressed in the intestine and it can promote fatty acid (FA) absorption. DGAT1 is also expressed in the testis, liver, and adipose tissues, while DGAT2 is highly expressed in the liver and adipose tissues, but it is expressed at low levels in the intestine [31]. Cytoplasmic Rip140 can interact with perilipin to regulate lipolysis [20]. In the current research, we found that both *DGAT1* and *DGAT2* expressions were upregulated, but RIP140 was downregulated in ccRCC tissues compared with those in the ANT tissues.

We determined whether there is an association between changes in these lipids‐associated genes and alterations in *RNASET2* expression levels. Our results documented that the expression of *DGAT1* and *DGAT2* decreased significantly in ccRCC cells when *RNASET2* was knocked down, whereas when *RNASET2* was overexpressed, the expression of *DGAT1* and *DGAT2* increased significantly. However, modulation of the *RNASET2* expression level did not affect changes in the *RIP140* expression levels. These results strongly confirmed that *RNASET2* can regulate the lipids‐synthesis genes *DGAT1* and *DGAT2* expression, which leads to increases in triglyceride synthesis and lipid droplet formation in ccRCC cells. Excess amounts of free fatty acids (FFAs) and diacylglycerols (DAGs) in the cell usually lead to lipotoxicity [32], while lipid droplets are generally considered as the central antilipotoxic organelles that control lipotoxicity by sequestering these potentially toxic lipids into inert triglycerides [32, 33, 34]. Herein, we speculate that *RNASET2* promoted the synthesis of TGs to resist lipotoxicity in ccRCC.

Moreover, we also investigated the possible pathways relevant to the function of RNASET2 protein in *VHL*‐deficient 786‐O cells through immunoprecipitation‐mass spectrometry (IP‐MS; Fig. S3). The results of IP‐MS showed that the proteins that interacted with RNASET2 were involved in various signaling pathways in 786‐O cells. Besides ribosome proteins, numerous other proteins are involved in the metabolic pathways especially in fatty acid metabolism in the MS list. They include fatty acid‐binding protein 5 (FABP5), elongation of very long‐chain fatty acid‐like 1 (ELOVL1), acyl‐CoA synthetase long‐chain family member proteins (ACSL3 and ACSL4), and metastasis‐associated protein MTA2. Lv *et al*. [35] reported that FABP5 expression was significantly upregulated in ccRCC and patients with a high expression level of FABP5 were correlated with tumor metastasis classifications that were also predicted to have poor survival. ELOVL1 is an enzyme elongating saturated and monounsaturated fatty acids [36]. Silencing *ELOVL1* can reduce the lipidomic profiles and viability of breast cancer cells [37]. In recent years, several studies demonstrated that ferroptosis was involved in the development of varied tumors. ACSL3 and ACSL4 can incorporate polyunsaturated fatty acids (PUFAs) into phospholipids, which is a critical step in ferroptosis [38]. ACSL3 is highly expressed in human lung cancer specimens and it can promote cancer initiation [39]. Oleic acid can protect melanoma cells from ferroptosis in an ACSL3‐dependent manner [40]. ACSL4 is upregulated in many cancers, including liver cancer, prostate cancer, breast cancer, and colon cancer [26]. In addition, MTA2 is a metastasis‐associated protein, which plays some vital roles during the metastasis of tumors [41, 42]. In the current study, there is a correlation between *MTA2* expression and *RNASET2* expression, as well as cancer cell migration in ccRCC cells. Accordingly, RNASET2 may have some special effects on the development and metastasis of ccRCC through interacting with these target proteins.

Hypoxic status is one of the crucial factors controlling malignant tumor development and metastasis. Uccella *et al*. [14] reported that the expression of the RNASET2 protein was induced in the *HIF1α*‐transfected human breast cancer‐derived MCF7 cell line. However, HIF1α activity is often deficient because of the chromosomal 14q loss in some ccRCC cases [43], whereas HIF2α is highly expressed in ccRCC due to *VHL* deficiency [24]. To investigate the mechanism involved in the regulation of *RNASET2* transcription, we performed ChIP experiments in the ccRCC cell line 786‐O characterized as having a *VHL* and *HIF1α* deficiency like most ccRCC cases. The ChIP‐PCR results showed that the transcription of *RNASET2* is upregulated by HIF2α directly, which is accumulated in *VHL*‐deficient ccRCC cells.

*VHL* is one of the most important tumor suppressor genes in humans. Some research documented that the *VHL* gene is a crucial factor in the pathogenesis of ccRCC. For instance, there are *VHL* mutations that include loss of chromosome 3p25 where the *VHL* gene is located, or promoter hypermethylation of the *VHL* gene in nearly 80% of ccRCC cases [44, 45, 46, 47]. *VHL* deficiency leads to the accumulation of HIF1α and HIF2α, which are hypoxia‐inducible factors. Although HIF2α has a far greater role in ccRCC, its role in tumorigenesis has been less extensively studied than that of HIF1α [48]. Our results employing TCGA database analysis also showed that there was a negative correlation between *RNASET2* and *VHL* expression levels in ccRCC cells. *VHL* deficiency‐induced HIF1α accumulation can be integrated with ARNT in the cells, and then, the integrated complex is transferred into the cell nucleus to activate the transcription of the target genes or downstream genes in the pathways. They include MMP9, VEGF, and PDGF, which can promote both cancer cell proliferation and metastasis [49, 50, 51]. Thus, most of the drugs applied in the clinics generally target the VHL/HIF1α/VEGF signaling pathway axes. For example, Sorafenib and Sunitinib are two drugs that have been approved for the therapeutic management of advanced‐stage renal cell cancer, which can depress the development of renal cell cancer by inhibiting VEGFR, PDGFR, and FLT3 expression levels [3, 52]. Bevacizumab, an anti‐VEGF recombination monoclonal antibody, can work together with interferon in treating ccRCC [53]. Besides, HIF2α is another critical therapeutic target for ccRCC treatment. Extensive biochemical efforts resulted in optimizing PT2385, a leading HIF2α inhibitor [54], which was later modified and improved and redesignated as PT2977, a second‐generation inhibitor (now known as ‘MK‐6482’) [55]. Both of these two inhibitors can block the transcription of HIF2α‐targeted genes, including *VEGFA*, *CCND1*, and *SLC2A1* [56, 57]. In the current study, we demonstrated that *RNASET2* is also the target gene of HIF2α in ccRCC. Therefore, it is suggested that the HIF2α inhibitors may also block the transcription of *RNASET2* gene.

In conclusion, the current research shows that overexpression of *RNASET2* is associated with increases in ccRCC progression, especially in poorly differentiated tumors. On the contrary, a negative correlation exists between *RNASET2* mRNA and *VHL* mRNA expressions. Meanwhile, increases in transcription factor HIF2α, in turn, upregulate *RNASET2* expression in ccRCC. Moreover, the results show that increases in *RNASET2* expression promote increases in gene expression of DGAT1 and DGAT2, which account for rises in both TGs syntheses. These effects ultimately protect the cancer cells and promote cell migration in ccRCC. Therefore, this research provides valuable insight for further etiological clarification of factors controlling ccRCC progression. This endeavor is likely to identify new therapeutic targets such as RNASET2 in the clinics. However, although we elucidated the signaling pathway through which changes in *RNASET2* expression levels modulate ccRCC oncogenesis *in vitro*, some important experiments *in vivo*, such as tumor xenograft in nude mice, are still needed to clarify how such changes affect ccRCC oncogenesis.

## Materials and methods

### Patient specimens and cell lines

This study enrolled subjects visiting the Department of Urology, Shanghai Ruijin Hospital between September 2018 and August 2021. Their clear cell renal cell carcinoma (ccRCC) tissues and adjacent normal tissues (ANT) were obtained during the surgical operation. The study conformed to the standards set by the Declaration of Helsinki was approved by the Ethics Committee of Ruijin Hospital, Shanghai Jiao Tong University School of Medicine, China (approval number: 2018‐381). All subjects gave written informed consent before their participation. Twenty‐eight pairs of samples including both ccRCC and ANT tissues were collected after surgery for the following experiments. ccRCC cell lines 786‐O and 769‐P and human proximal tubular epithelial cell line HK‐2 were provided by the National Collection of Authenticated Cell Cultures. 786‐O cells and 769‐P cells were immediately cultured in RPMI1640 medium (Gibco, Waltham, MA, USA) and HK‐2 cells were cultured in DME/F12 medium (Gibco) when the cell lines were obtained. All the culture media contained 10% fetal bovine serum (FBS), 100 U·mL^−1^ penicillin, and 100 μg·mL^−1^ streptomycin, and the cultured cells were kept in 95% humidity and 37 °C 5% CO_2_ incubator (Thermo, Waltham, MA, USA).

### RNA isolation and quantitative Real‐Time PCR

Total RNA was extracted with the TRIzol reagent (Invitrogen, Carlsbad, CA, USA). cDNA synthesis of genes was performed with a PrimeScript™ RT Master Mix (Takara, Shiga, Japan). Subsequently, quantitative real‐time PCR was carried out with TB Green® Premix Ex Taq™ (Tli RNaseH Plus) mix (Takara). The PCR system was successfully run in Applied Biosystem 7500 (ABI, Waltham, MA, USA). All the primers for real‐time RT‐PCR analyses were designed by primer premier 6 software (Premier Inc., Palo Alto, CA, USA), which is shown in Table S1.

### Protein purification and western blot

Tissues or cells were lysed using RIPA lysis buffer supplemented with protease inhibitors (Yeasen, Shanghai, China). Equal amounts of total protein lysate (30 μg per sample) were separated by 4 ~ 12% SDS/PAGE and then were transferred onto a PVDF membrane (Millipore, Billerica, MA, USA) by a wet transfer apparatus (Bio‐Rad, Hercules, CA, USA). After that, the membranes were blocked with 5% BSA in TBS‐T (Tris–HCl/NaCl solution with 1‰ tween 20) and probed with primary antibodies (dilution 1 : 1000) overnight at 4 °C (RNASTE2: ab107313, Abcam, Cambridge, UK; HIF2α: NB100‐122, Novus, Centennial, CO, USA) while β‐actin (cat: 4967, CST, Beverly, MA, USA) was used as the internal control (dilution 1 : 1000), followed by incubation of corresponding horseradish peroxidase‐linked secondary antibody (CST) for 2 h at room temperature. Finally, the membranes were reacted with ECL western blot substrate kit (Millipore) before exposure with ImageQuant LAS4000 (GE, Chicago, IL, USA).

### Immunohistochemistry (IHC) staining

IHC staining was performed after dewaxing and rehydrating paraffin‐embedded tissue sections. Subsequently, antigen retrieval was done by boiling the tissue for 15 min in 10 mm citrate buffer, pH 6.0. Then, the Histostain LAB‐SA Detection kits (Invitrogen, OR, USA) were applied according to the manufacturer's instructions, and the sections were stained using DAB, while nuclei were counterstained with hematoxylin. After the sample was washed with PBS buffer thrice, the digital images were captured under a microscope (Olympus BX53) equipped with a DP74 40× objective (Olympus, Tokyo, Japan).

### Oil red O staining

The Oil Red O staining was followed by the Modified Oil Red O staining kit protocol (Solarbio, Beijing, China). Briefly, the frozen sections or cell slides were fixed in 10% formalin for 10 min and washed with distilled water, and then immersed in 60% isopropanol for 30 s. Then, the slides were immersed into the modified Oil Red O staining buffer immediately for 10 min. Next, the superfluous staining buffer was removed, and then, the slides were washed with 60% isopropanol and distilled water, respectively. Meanwhile, the nuclei were counterstained with hematoxylin for 1 min and washed with distilled water. Finally, the slides were sealed with gelatin and the digital images were captured under a microscope (Olympus) equipped with a DP74 40× objective (Olympus).

### Lipi‐red staining

Lipi‐red staining (DOJINDO, Kumamoto, Japan) evaluated the extent of lipid droplet accumulation. G418 (200 μg·mL^−1^) exposure was performed to screen for cell stability. The cells were plated and expanded on slides until they reached a density of about 70% at 37 °C and 5% CO_2_. One micromolar lipi‐red work solution was added and incubated at 37 °C 5% CO_2_ for 30 min. The cells were washed with PBS thrice and then covered with the antifade mounting solution containing DAPI (Yeasen). The pictures were captured under a microscope (Olympus).

### Cell viability assay

The Cell Counting Kit‐8 (CCK‐8; DOJINDO) evaluated the effect of *RNASET2* knockdown on 786‐O cell viability. Stable screening cells were isolated by exposure to 200 μg·mL^−1^ G418. The cells were expanded until they reached a cell density of about 70% at 37 °C 5% CO_2_. CCK‐8 (5 mg·mL^−1^) was added and the cells were incubated at 37 °C under 5% CO_2_ for 1 h. Optical density (OD) was read on a microplate reader at an absorbance value of 450 nm (Thermo). Three independent experimental replicates were performed.

### Transmission electron microscopy (TEM) analysis

Tissues for TEM were prepared just following the protocols [58]. Briefly, small pieces of ccRCC tissues and ANT tissues were immersed in 2.5% glutaraldehyde solubilized in 0.1 m phosphate buffer (pH 7.4) for 1 day. The tissues were then fixed in 1% osmium tetroxide and dehydrated through a graded ethanol series, and embedded in Epon 618 (TAAB Laboratories Equipment, Berks, UK). Ultra‐thin sections (70 ~ 90 nm) sliced from the tissues were stained with lead citrate and uranyl acetate and then examined at 100 kV with a Philips CM‐120 (Philips, Eindhoven, the Netherlands).

### shRNA plasmids transfection

To construct shRNA plasmids, the shRNA oligo for *shRNASET2* (forward sequence 5′‐GCAAGAGAAAUUCACAAACUGCAGC‐3′ and reverse sequence 5′‐GCUGCAGUUUGUGAAUUUCUCUUGCUU‐3′) and control shRNA (forward sequence 5′‐CUUCCUCUCUUUCUCUCCCUUGUGA‐3′ and reverse sequence 5′‐UCACAAGGGAGAGAAAGAGAGGAAGGA‐3′) [28] were cloned into the pGPU6/GFP/Neo vector (GenePharma, Shanghai, China) according to the manufacturer's instruction. ccRCC cell line 786‐O cells or 769‐P cells were transfected with *RNASET2* shRNA plasmids or control shRNA plasmids using HighGene transfection reagent (Abclonal, Wuhan, China) and selected for 2 weeks in 500 μg·mL^−1^ Geneticin (G418; Selleck, Houston, TX, USA). Subsequently, the selected cells were cultured in 200 μg·mL^−1^ G418.

### Real‐Time cell analysis (RTCA)

A real‐time cell analysis experiment is a more accurate method to estimate cell states using micro‐electronic biosensor technology. This method detects time‐dependent changes in cell proliferation in real time. Briefly, 50 μL of cell culture medium was added to each well of the RTCA plate to establish a baseline. Then, 100 μL of a cell suspension containing about 5000 cells was added to each well. Finally, cells were auto‐scanned using RTCA xCELLigence system (ACEA Biosciences, San Diego, CA, USA) every 15 min.

### Scratch wound healing assay

To measure cell migration, a scratch wound healing assay was performed. ccRCC cells transfected with *RNASET2* shRNA‐pGPU6/GFP/Neo plasmids and scramble sequence‐pGPU6/GFP/Neo plasmids (control) were planted in 6‐well plates with horizontal lines predrawn on the plate back, respectively. Cells grew to form a monolayer at 37 °C 5% CO_2_ under normoxic conditions overnight. Subsequently, cells were removed from the plates by scratching their surfaces with 10 μL pipette tips to create vertical wounds perpendicular to the drawn horizontal lines. Wells were washed thrice with PBS to remove the floating cells, and then, fresh RPMI1640 medium without FBS was added to the wells. The transfected cells 786‐O and 769‐P were incubated at 37 °C, 5% CO_2_ conditions for 15 and 31 h, respectively, depending on their migration rate. Then, the photographs were captured under the inverted microscope (Olympus). Five fields were randomly selected and the scratch wound healing was analyzed with imagej software (NIH, Bethesda, MD, USA).

### Transwell assay

The upper transwell chamber was coated with a cell suspension containing 10 × 10^4^·mL^−1^ cells and Matrigel 200 μL with serum‐free RPMI‐1640 medium was added to this chamber, and 600 μL RPMI‐1640 medium containing 20% fetal bovine serum was added to the lower chamber, respectively. The upper chamber was carefully immersed in the lower chamber liquid. A plate consisting of 24‐well transwell chambers was incubated at 37 °C under 5% CO_2_ for 24 h. The liquid was removed from the upper chamber and washed thrice with 600 μL PBS. After staining, the upper chamber with crystal violet was observed under a microscope and photographed (Olympus). Three independent experimental replicates were performed.

### CoCl_2_ treatment

ccRCC cell line 786‐O cells or 769‐P cells were planted in a 6‐well plate and incubated at 37 °C, 5% CO_2_ overnight. Then, the cells were incubated with fresh RPMI1640 media containing 10% FBS and treated with 0, 50, 100, 150, and 300 μm CoCl_2_, respectively, for 24 h. Cells were harvested and washed thrice with PBS buffer, and then applied for use in various experiments.

### PT2385 treatment

Cells were placed in a 6‐well plate and incubated at 37 °C under 5% CO_2_ overnight. Then, the medium was replaced with fresh RPMI1640 medium containing 10% FBS and exposed to 0.01, 0.1, 1.0, and 10 μm PT2385, respectively, for 24 h (control: DMSO). Cells were harvested and washed thrice with PBS buffer and then applied for use in various experiments.

### Coimmunoprecipitation and mass spectrometry (MS)

For MS analysis, coimmunoprecipitation was performed according to Capturem™ IP & Co‐IP Kit (Takara). Briefly, 786‐O cells were washed with cold PBS once the culture media was removed. The cellular lysate was prepared using lysis/equilibration buffer and incubated on ice for 15 min and then centrifuged at 17 000 × ***g*** for 10 min at 4 °C. The supernatant was transferred into a new 1.5 mL tube and incubated with RNASET2 antibody on a rotator at 4 °C overnight. Next, 100 μL of lysis buffer was added to a spin column in a provided collection tube for equilibration, and then, the tube was centrifuged at 1000 × ***g*** for 1 min at room temperature. The column was placed into a new collection tube following loading of the protein‐RNASET2 antibody mixture and centrifuged at 1000 × ***g*** for 1 min at room temperature. Finally, the sample flow‐through was saved for MS analysis. The MS analysis was performed with the Orbitrap Fusion Lumos spectrometer (ThermoFisher Scientific). The peak list MS features were annotated using KEGG (Kyoto Encyclopedia of Genes and Genomes) pathway analysis.

### Chromatin immunoprecipitation‐PCR (ChIP‐PCR)

786‐O cells were cross‐linked with formaldehyde, and chromatin was isolated and sheared by the Bioruptor Sonication System (Diagenode, Liege, Belgium) to obtain an average fragment size of 200 ~ 1000 bp. Sheared chromatin fragments were incubated with the anti‐HIF2α antibody (NB100‐122; Novus). Meanwhile, an equal concentration of nonspecific IgG was used as the negative control. The immunoprecipitated protein‐DNA complex was obtained using protein A/G immunomagnetic beads, and DNA was isolated by the standard phenol/chloroform/ethanol precipitation method. PCR was amplified with the *RNASET2* primers (forward primer 5′‐GGTGGTCTAGGTTAGCTCGG‐3′; reverse primer: 5′‐GCTGTGTTCCTTCGCCAC‐3′). The amplicon was corresponding to the −278 to −187 region located the upstream of *RNASET2* transcript start site (TSS).

### Statistical analysis

All data were analyzed using the sas 8.2 software (SAS Institute Inc., Cary, NC, USA), and results are presented as mean ± SD. The paired *t*‐test or Student's *t*‐test was used where appropriate. A one‐way analysis of variance test was used assuming a two‐tailed hypothesis with *P* < 0.05. The survival curves were plotted using the Kaplan–Meier method, and the differences were assessed using the log‐rank test. *P* < 0.05 was considered a statistically significant difference. The gray values of western blot bands were analyzed by imagej software (NIH). In addition, the survival time of subjects was calculated from the date of surgery to the date of death. Statistical analysis of survival time was carried out using the Kaplan–Meier survival test. Meanwhile, the cancer genome atlas program (TCGA) and the online tool gepia2 (gepia2.cancer‐pku.cn) were also applied to analyze the survival curve.

## Conflict of interest

The authors declare no conflict of interest.

## Author contributions

YQ, LZ, LJ, YLo, and YH performed all the experiments. JD, SZ, and SL contributed to reagents and material preparation. YQ analyzed the data and wrote the manuscript. ZD, JZ, and YLi contributed to the planning and supervising of the study and revision of the manuscript.

## Supporting information


**Table S1.** Primer sequences for qPCR.

**Fig. S1.** Effects of *RNASET2* knockdown or overexpression on ccRCC cells. (A) RNASET2 knockdown in 786‐O cells had no significant effect on the expression of lipolysis‐related genes, *n* = 6; (B) Viability of 786‐O cells was evaluated with the CCK‐8 kit. The results showed that RNASET2 shRNA transfection suppressed this parameter, *n* = 4; (C) RNASET2 knockdown downregulated metastasis‐associated genes MTA2 in 786‐O cells, *n* = 6; (D) RNASET2 overexpression in 769‐P cells upregulated the expressions of DGAT1 and DGAT2 but did not influence RIP140 expression, *n* = 6; (E) RNASET2 overexpression in 769‐P cells did not influence cell proliferation. Values in bar graphs are the mean with SD. Statistical analysis was performed using the Student's *t*‐test. *P < 0.05, **P < 0.01, ****P < 0.0001; ns, not significant.

**Fig. S2.** Protein levels in ccRCC tissues and ANT. (A) Western blot analysis showed that the HIF1α protein was deficient in ccRCC tissues; (B) Western blot analysis showed that the HIF2α protein expression level was higher in ccRCC tissues than that in ANT.

**Fig. S3.** Results of coimmunoprecipitation and MS: RNASET2 antibody pulled down proteins in 786‐O cells. KEGG pathway analysis, the arrow points to metabolic pathways.

## Data Availability

The data that support the findings of this study are available upon request from the corresponding author. The data are not publicly available due to privacy or ethical restrictions.
