# Supplementary material for: HIF2α‐induced upregulation of RNASET2 promotes triglyceride synthesis and enhances cell migration in clear cell renal cell carcinoma
Source: FEBS Open Bio. 2023 Feb 12;13(4):638–54. doi: 10.1002/2211-5463.13570 (PMC10068329; doi:10.1002/2211-5463.13570)
Supplement: Supplementary file 1 — Table S1. Primer sequences for qPCR. [file FEB4-13-638-s004.docx]

**Table S1 Primer sequences for qPCR**

| Gene Symbol | sequences | |
| --- | --- | --- |
| *RNASET2* | Forward | 5’- ACCCTCCGGATTACTGGACA-3’ |
|  | Reverse | 5’- TGCGATTGGGAAACGAGTGA-3’ |
| *DGAT1* | Forward | 5’- TGAGCGTCCCTCTGCGAATGT-3’ |
|  | Reverse | 5’- CGATGATGAGCGACAGCCACAC-3’ |
| *DGAT2* | Forward | 5’- GGAGGTCACAGTGGGTCCGAAA-3’ |
|  | Reverse | 5’- GGAAGTTGCCTGCCAGTGTAGC-3’ |
| *MTA2* | Forward | 5’- GGACCCAGACAACCCTCTCACA-3’ |
|  | Reverse | 5’- TTAGCCAGGTCGTAGCCGTTCC-3’ |
| *ACTB* | Forward | 5’- GCACGGCATCGTCACCAACT-3’ |
|  | Reverse | 5’- CCAGAGGCGTACAGGGATAGCA-3’ |
