# Supplementary figures and images for: HIF2α‐induced upregulation of RNASET2 promotes triglyceride synthesis and enhances cell migration in clear cell renal cell carcinoma
Source: FEBS Open Bio. 2023 Feb 12;13(4):638–54. doi: 10.1002/2211-5463.13570 (PMC10068329; doi:10.1002/2211-5463.13570)

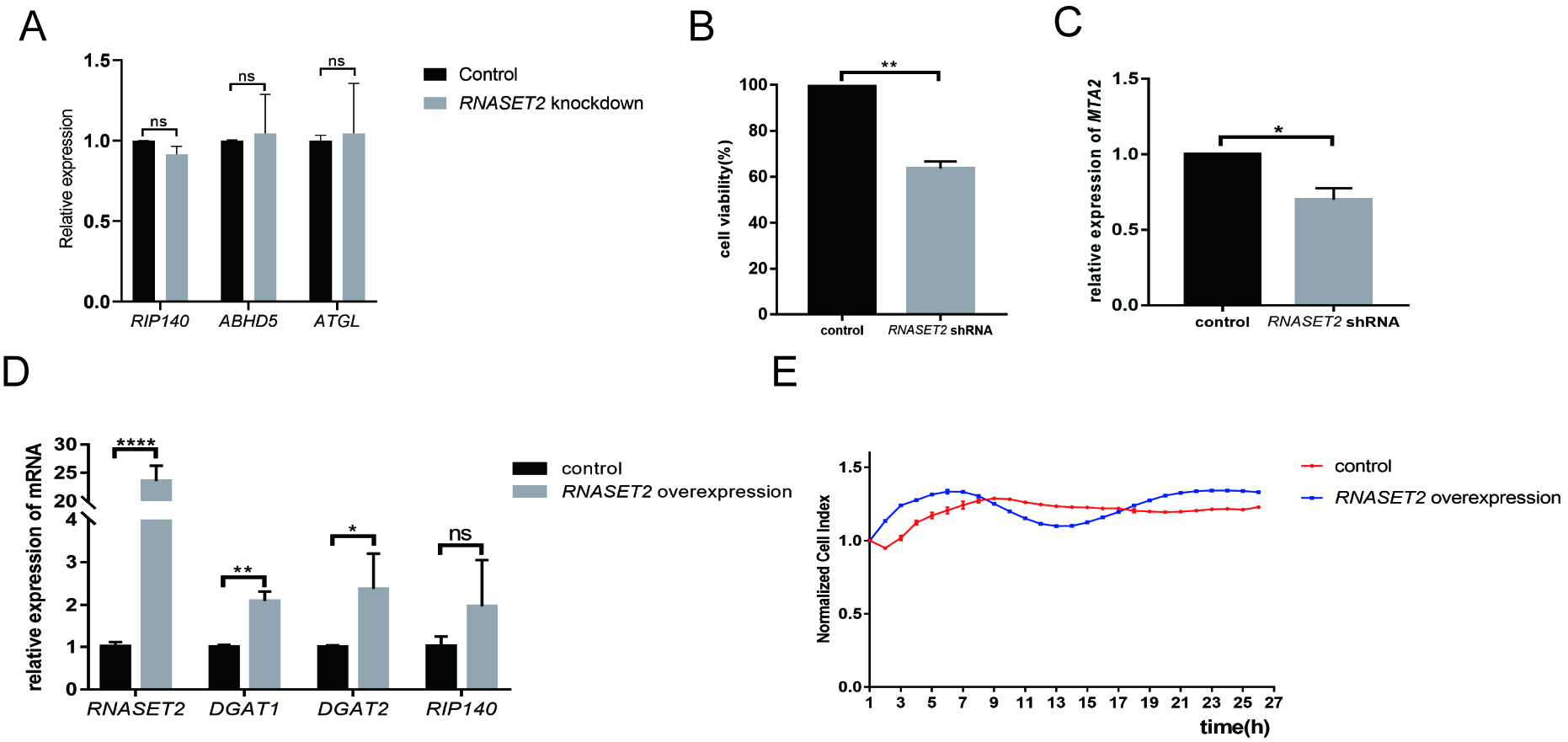

Supplement: Supplementary file 2 — Fig. S1. Effects of RNASET2 knockdown or overexpression on ccRCC cells. (A) RNASET2 knockdown in 786‐O cells had no significant effect on the expression of lipolysis‐related genes, n = 6; (B) Viability of 786‐O cells was evaluated with the CCK‐8 kit. The results showed that RNASET2 shRNA transfection suppressed this parameter, n = 4; (C) RNASET2 knockdown downregulated metastasis‐associated genes MTA2 in 786‐O cells, n = 6; (D) RNASET2 overexpression in 769‐P cells upregulated the expressions of DGAT1 and DGAT2 but did not influence RIP140 expression, n = 6; (E) RNASET2 overexpression in 769‐P cells did not influence cell proliferation. Values in bar graphs are the mean with SD. Statistical analysis was performed using the Student's t‐test. *P < 0.05, **P < 0.01, ****P < 0.0001; ns, not significant. [file FEB4-13-638-s005.tif]

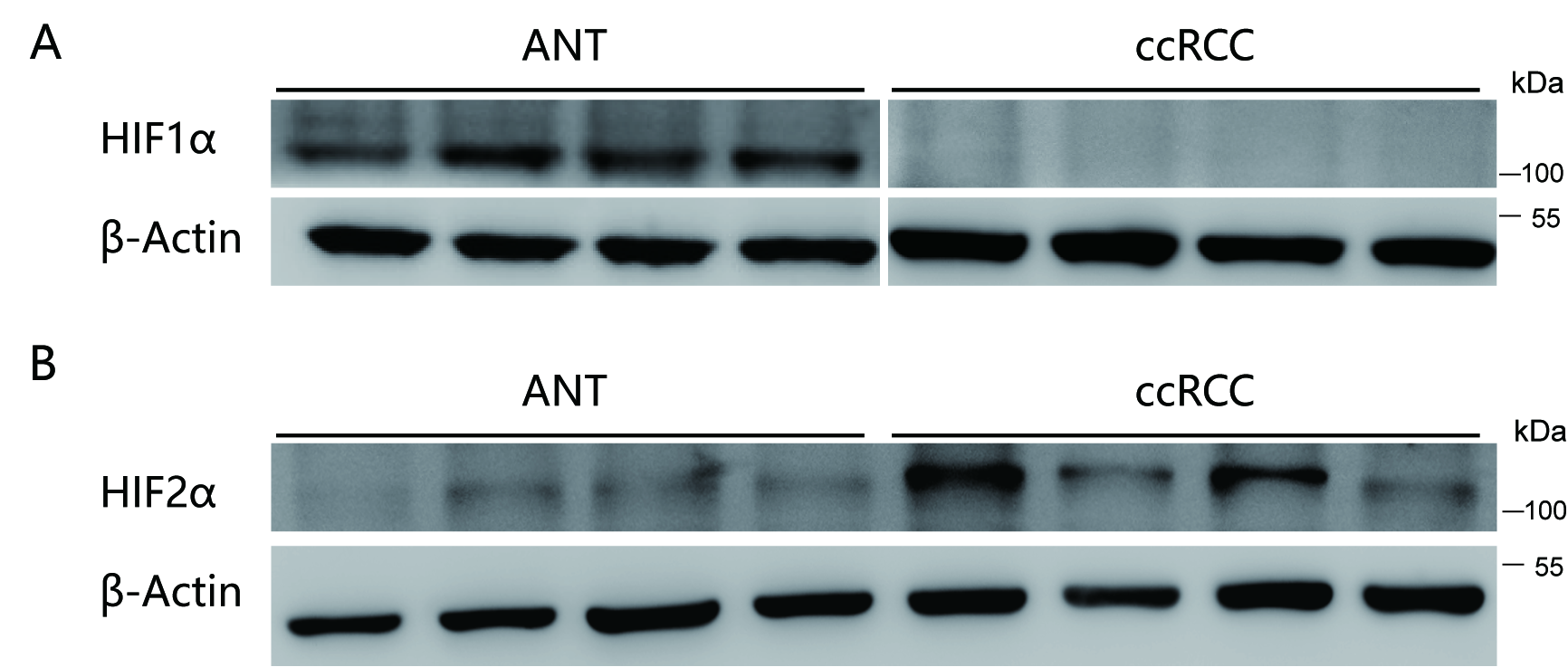

Supplement: Supplementary file 3 — Fig. S2. Protein levels in ccRCC tissues and ANT. (A) Western blot analysis showed that the HIF1α protein was deficient in ccRCC tissues; (B) Western blot analysis showed that the HIF2α protein expression level was higher in ccRCC tissues than that in ANT. [file FEB4-13-638-s003.tif]

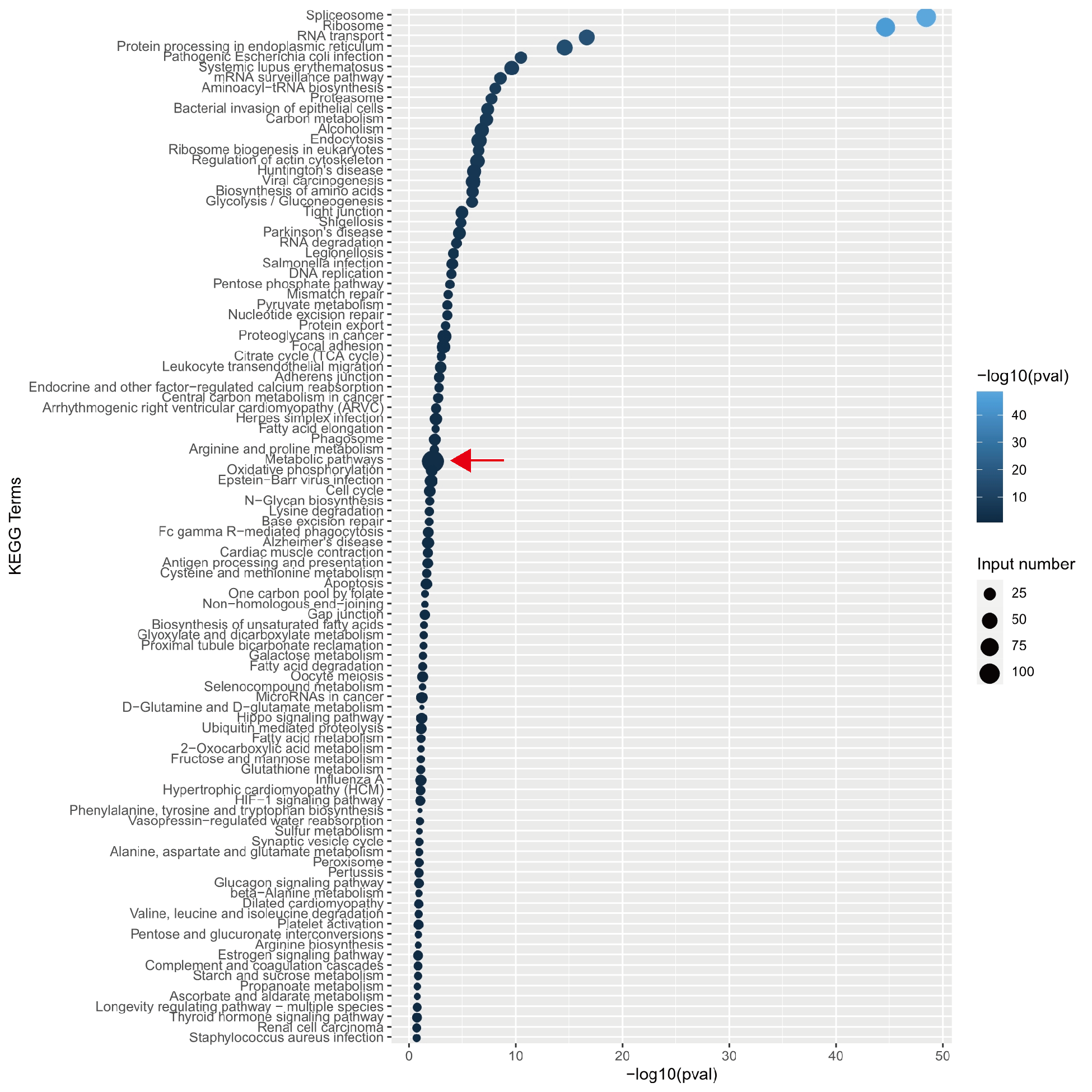

Supplement: Supplementary file 4 — Fig. S3. Results of coimmunoprecipitation and MS: RNASET2 antibody pulled down proteins in 786‐O cells. KEGG pathway analysis, the arrow points to metabolic pathways. [file FEB4-13-638-s001.tif]
