# Supplementary material for: HIF2α‐induced upregulation of RNASET2 promotes triglyceride synthesis and enhances cell migration in clear cell renal cell carcinoma
Source: FEBS Open Bio. 2023 Feb 12;13(4):638–54. doi: 10.1002/2211-5463.13570 (PMC10068329; doi:10.1002/2211-5463.13570)
Supplement: Supplementary file 5 [file FEB4-13-638-s002.docx]

**Supplementary tables/figures**

**Table S1. Primer sequences for qPCR**

**Figure S1. Effects of *RNASET2* knockdown or overexpression on ccRCC cells.** **A**, *RNASET2* knockdown in 786-O cells had no significant effect on the expression of lipolysis-related genes, n = 6; **B**, Viability of 786-O cells was evaluated with the CCK-8 kit. The results showed that *RNASET2* shRNA transfection suppressed this parameter, n = 4; **C**, *RNASET2* knockdown downregulated metastasis-associated genes *MTA2* in 786-O cells, n = 6; **D**, *RNASET2* overexpression in 769-P cells upregulated the expressions of *DGAT1* and *DGAT2*, but did not influence *RIP140* expression, n = 6; **E**, *RNASET2* overexpression in 769-P cells did not influence cell proliferation. Values in bar graphs are the mean with SD. Statistical analysis was performed using Student’s *t*-test. **p*<0.05, ***p*<0.01, *****p*<0.0001; ns, not significant.

**Figure S2. Protein levels in ccRCC tissues and ANT. A**, Western blot analysis showed that the HIF1α protein was deficient in ccRCC tissues; **B**, Western blot analysis showed that the HIF2α protein expression level was higher in ccRCC tissues than that in ANT.

**Figure S3. Results of Co-immunoprecipitation and MS: RNASET2 antibody pulled down proteins in 786-O cells.** KEGG pathway analysis, the arrow points to metabolic pathways.
